# Aquaporin-3 and Aquaporin-5 Facilitate Migration and Cell–Cell Adhesion in Pancreatic Cancer by Modulating Cell Biomechanical Properties

**DOI:** 10.3390/cells11081308

**Published:** 2022-04-12

**Authors:** Patrícia M. Silva, Inês V. da Silva, Maria J. Sarmento, Ítala C. Silva, Filomena A. Carvalho, Graça Soveral, Nuno C. Santos

**Affiliations:** 1 Instituto de Medicina Molecular, Faculdade de Medicina, Universidade de Lisboa, 1649-028 Lisbon, Portugal; patriciamsilva@medicina.ulisboa.pt (P.M.S.); maria.sarmento@medicina.ulisboa.pt (M.J.S.); itala.silva@medicina.ulisboa.pt (Í.C.S.); filomenacarvalho@medicina.ulisboa.pt (F.A.C.); 2 Research Institute for Medicines (iMed.ULisboa), Faculty of Pharmacy, Universidade de Lisboa, 1649-003 Lisbon, Portugal; imsilva1@campus.ul.pt; 3 Department of Pharmaceutical Sciences and Medicines, Faculty of Pharmacy, Universidade de Lisboa, 1649-003 Lisbon, Portugal

**Keywords:** aquaporins, aquaglyceroporins, hydrogen peroxide, pancreatic cancer, cell–cell adhesion, membrane fluidity, atomic force microscopy

## Abstract

Background: Aquaporins are membrane channels responsible for the bidirectional transfer of water and small non-charged solutes across cell membranes. AQP3 and AQP5 are overexpressed in pancreatic ductal adenocarcinoma, playing key roles in cell migration, proliferation, and invasion. Here, we evaluated AQP3 and AQP5 involvement in cell biomechanical properties, cell–cell adhesion, and cell migration, following a loss-of-function strategy on BxPC-3 cells. Results: Silencing of AQP3 and AQP5 was functionally validated by reduced membrane permeability and had implications on cell migration, slowing wound recovery. Moreover, silenced AQP5 and AQP3/5 cells showed higher membrane fluidity. Biomechanical and morphological changes were assessed by atomic force microscopy (AFM), revealing AQP5 and AQP3/5 silenced cells with a lower stiffness than their control. Through cell–cell adhesion measurements, the work (energy) necessary to detach two cells was found to be lower for AQP-silenced cells than control, showing that these AQPs have implications on cell–cell adhesion. Conclusion: These findings highlight AQP3 and AQP5 involvement in the biophysical properties of cell membranes, whole cell biomechanical properties, and cell–cell adhesion, thus having potential implication in the settings of tumor development.

## 1. Introduction

The transport of water and solutes across membranes is of utmost importance in human physiology. Aquaporins (AQPs) are a class of transmembrane channels that facilitate the bidirectional flow of water and small neutral solutes driven by osmotic and solute gradients, respectively [1,2]. AQPs are widely expressed in all kinds of organisms and cells. In humans, the thirteen described paralogs (AQP0-12) are expressed in a cell- and tissue-dependent pattern [3], each of them presenting different features. According to their permeability and primary structure, AQPs were categorized in three sub-groups: classical or orthodox AQPs (AQP0, AQP1, AQP2, AQP4, AQP5, AQP6 and AQP8) are primarily selective to water and play an important role in fluid transport-specialized tissues, such as epithelia; aquaglyceroporins (AQP3, AQP7, AQP9 and AQP10) are also permeable to small non-charged solutes, such as glycerol and urea, thus impacting glycerol metabolism and energy balance; and, unorthodox or S-aquaporins (AQP11 and AQP12) are intracellular paralogs that regulate organelle homeostasis [4,5,6]. Recently, several aquaporins (AQP0, AQP1, AQP3, AQP5, AQP8, AQP9, and AQP11) were reported for their role in hydrogen peroxide (H_2_O_2_) transport, and so termed peroxiporins [7,8,9]. H_2_O_2_ is the major reactive oxygen species (ROS), and cell physiology maintenance is H_2_O_2_ intracellular concentration-dependent. In fact, at low intracellular concentrations (1–10 nM), H_2_O_2_ acts as a secondary messenger with a crucial role in redox signaling, maintaining the cell physiology; however, at higher concentrations (above 10 nM), H_2_O_2_ oxidizes cellular components, such as DNA, proteins and lipids, and disrupts the finely regulated redox signaling, leading to oxidative stress [10]. Such oxidant environments promote inflammation, tumor growth, and metastasis, triggering the development of malignant tumors [11]. One of the mechanisms by which aquaporins play an important role in cancer is related to their ability to fine-tune H_2_O_2_ membrane permeability impacting cell proliferation, differentiation, and apoptosis [12,13].

Pancreatic cancer (PC) is one of the most aggressive digestive tumors, the seventh most common cancer worldwide, and the third leading cause of death in males and the fourth in females from oncologic cause in the EU. Pancreatic ductal adenocarcinoma (PDA) is the most frequent and the most lethal PC [14]. Despite novel pharmacologic agents coming out over the last decade, targeted therapy has little efficacy and surgical resection is still the best chance for long-term survival, even with high recurrence rates and poor outcomes [15]. New anticancer strategies are therefore an increasing priority.

In a previous study, AQP3 and AQP5 expression was investigated in pancreatic ductal adenocarcinoma (PDA) human biopsies by immunohistochemistry [16] and, although both AQPs have peroxiporin activity [9], their expression pattern was different during the development of the tumor. AQP3 expression increased along the development of PDA, from the early to later stages of disease, while AQP5 was increased in the early stages and almost undetectable in later stages, suggesting AQP5 as a promising biomarker for PDA early diagnosis. AQP3 and AQP5 were also correlated with increased levels of epidermal growth factor receptor (EGFR), proliferation marker protein Ki-67 (Ki-67), cytokeratin 7 (CK7), and vimentin (Vim), as well as decreased E-caderin (E-cad) [16], suggesting contribution to the epithelial-mesenchymal transition (EMT) process, tumor formation, and invasion. Such findings suggest peroxiporins as key players in the maintenance of redox balance, opening new perspectives for the development of innovative therapies for oxidative stress-related disorders, including cancer [17,18].

AQPs play fundamental roles in several other biological processes that induce tumor development and spread [19,20,21]. In gastric cancer, AQP3 correlates with EMT-related proteins, modulating cell proliferation, migration, and invasion in vitro, also inducing E-cad repression [19]. Furthermore, AQP-mediated water fluxes promote actin polymerization and stabilization, enhancing the formation of cell membrane protrusions and the dynamics of cell motility [22]. Additionally, by facilitating the permeation of several molecules, such as glycerol and H_2_O_2_, and interacting with oncoproteins, AQPs are postulated to activate intracellular signaling cascades that promote the transcription of key genes involved in tumor cell division and proliferation [4,21,23].

Taking into consideration the increased levels and the differential expression of AQP3 and AQP5 in PDA biopsies, we used a loss-of-function strategy to evaluate the effect of these two AQPs in tumoral features of BxPC-3 cells, an in vitro model of PDA. The model was initially validated by assessing transcript levels by quantitative polymerase chain reaction (qPCR) and immunoblot, respectively. Functional validation was achieved by measurements of water, glycerol, and H_2_O_2_ permeability by fluorescence microscopy techniques. Then, the impact of silencing AQP3 and/or AQP5 in biophysical, biomechanical, and morphological properties of pancreatic cancer cells was investigated by atomic force microscopy (AFM) and two-photon microscopy (TPM). Moreover, AQP3- and AQP5-silencing effect was evaluated in cell migration, cell–cell adhesion, and apoptosis processes.

## 2. Materials and Methods

### 2.1. Cell Culture

BxPC-3 cell line was obtained from ATCC (catalog no. CRL-1687) and cultured at 37 °C in 5% CO_2_. Cells were grown in RPMI1640 medium (Gibco, Thermo Fisher Scientific, Waltham, MA, USA) supplemented with 10% (*v*/*v*) heat-inactivated Fetal Bovine Serum (FBS; Gibco, Thermo Fisher Scientific), and 1% (*v*/*v*) penicillin/streptomycin (PenStrep, Invitrogen, Thermo Fisher Scientific). Experiments were performed with 70–80% cell confluence.

### 2.2. Transfection of BxPC-3 Cells

For AQPs knock-down, small interfering RNA (siRNA)-containing expression vectors targeting human AQP3 and AQP5 (ID: s1521 and ID: s1527, respectively, Ambion, Thermo Fisher Scientific) combined with Lipofectamine RNAiMAX Reagent (Invitrogen) were used according to the manufacturer protocol. A control of silencing (Silencer Negative Control siRNA #1, Ambion, Thermo Fisher Scientific) was also performed in parallel. For cell transfection, cells were seeded with an inoculum of 20,000 cells/cm^2^ in 6- and 12-well plates, according to each experiment needs. Transfections were validated by qPCR and functional assays. Cells were used for experiments 48 h post-transfection. Experimental groups were stablished as follows: BxPC-3 cells transfected with negative control (siControl), BxPC-3 cells transfected with AQP3 siRNA (siAQP3), BxPC-3 cells transfected with AQP5 siRNA (siAQP5), and BxPC-3 cells transfected with both AQP3 and AQP5 siRNAs (siAQP3/5).

### 2.3. RNA Isolation and RT-PCR

Total RNA was isolated from samples with 2 × 10^6^ cells using Trizol (Thermo Fisher Scientific), and extraction was performed according to manufacturer instructions. First strand cDNA was synthesized from 1 μg total RNA with quality ratios 260/280 and 260/230 (from 1.8 to 2.2, NanoDrop1 ND-2000c, ThermoFisher Scientific) and the reverse transcription was carried out using NZYFirst-strand cDNA synthesis kit (NZYtech, Lisbon, Portugal). Transcript levels for all AQPs paralogs (AQP0-12) and respective housekeeping gene HPRT-1 were quantified by real-time quantitative PCR (RT-PCR) using TaqMan Universal Master Mix II with UNG (Thermo Fisher Scientific) and following human-specific predesigned TaqMan Gene Expression Assays: AQP0 (Hs0085175_m1), AQP1 (Hs01028916_m1), AQP2 (Hs00166640_m1), AQP3 (Hs01105469_g1), AQP4 (Hs00242342_m1), AQP5 (Hs00387048_m1), AQP6 (Hs00155808_m1), AQP7 (Hs00357359_m1), AQP8 (Hs01086280_g1), AQP9 (Hs00175573_m1), AQP10 (Hs00369738_m1), AQP11 (Hs005426181_m1), AQP12 (Hs01651303_m1), and HPRT-1 (Hs02800695_m1). Gene expression evaluations were performed in a CFX96 RT-PCR Detection System C1000 (BioRad, Hercules, CA, USA). Data was normalized using housekeeping gene values, and relative quantification was calculated using a variation of the Livak method [24], described by Fleige and Pfaffl [25]. All samples were run in triplicate.

### 2.4. Water and Glycerol Permeability

Water (P_f_) and glycerol (P_gly_) permeability were measured in individual adherent cells on coverslips, as previously described [26]. Briefly, 48 h post-transfection, cells were loaded with 5 mM calcein acetoxymethyl ester (calcein-AM; Sigma-Aldrich, St. Louis, MO, USA) for 30 min at 37 °C in 5% CO_2_. Coverslips with the adherent cells were mounted on a perfusion chamber (Warner Instruments, Hamden, CT, USA) on the stage of a Zeiss Axiovert 200 inverted microscope (Jena, Germany). Fluorescence was recorded with excitation at 495 nm, with 10 nm bandwidth, and emission collected with a 535/25 nm bandpass filter coupled with a 515 nm dichroic beam splitter. Images were captured using 40× magnification and a digital camera (CoolSNAP EZ, Photometrics, Tucson, AZ, USA), and recorded using the Metafluor software (Molecular Devices, Sunnyvale, CA, USA). Cells were perfused with 300 mM HEPES buffer (135 mM NaCl, 5 mM KCl, 2.5 mM CaCl_2_, 1.2 mM MgCl_2_, 10 mM d-glucose, 5 mM HEPES, pH 7.4, 300 mOsM) for 40 s, after which 300 mM mannitol (for water permeability) or 300 mM glycerol (for glycerol permeability) was added to the buffer, to achieve an external osmolarity of 600 mOsM. Cell volume (V) was measured at selected time points from 2D images obtained during the permeability assays to evaluate the initial volume (prior to the osmotic challenge, V_o_) and the final equilibrium volume [27]. P_f_ and P_gly_ coefficients were evaluated from the measured time-dependent volume changes, V_rel_ = V/V_o_, obtained by adding mannitol (P_f_) or glycerol (P_gly_) to the external media, using the model equations described by Madeira et al. [28] and the Berkeley Madonna software (http://www.berkeleymadonna.com accessed on 10 February 2022).

### 2.5. Hydrogen Peroxide Influx

To evaluate H_2_O_2_ influx, oxidation kinetics of 2′,7′-dichlorodihydrofluorescein diacetate (H_2_-DCFDA, Invitrogen) were measured after challenging cells with H_2_O_2_, according to previously described methods [29]. BxPC-3 cells were seeded in coverslips at a density of 20,000 cells/cm^2^ prior to transfection. After 48 h of incubation with silencing reagents, H_2_O_2_ influx was indirectly measured in individual adherent cells on a coverslip mounted in a closed chamber (Warner Instruments) on the stage of a Zeiss Axiovert 200 inverted microscope, using a 40× epifluorescence objective. Fluorescence was recorded with excitation at 495 nm, with 10 nm bandwidth, and emission collected with a 515/10 nm bandpass filter. Data were recorded and analyzed using the Metafluor software (Molecular Devices), connected to a CCD camera (Cool SnapTM EZ, Photometrics). Briefly, cells were loaded with 10 µM H_2_-DCFDA for 30 min at 37 °C in 5% CO_2_. Fluorescence signal was acquired for cells in HEPES buffer (135 mM NaCl, 5 mM KCl, 2.5 mM CaCl_2_, 1.2 mM MgCl_2_, 10 mM d-glucose, 5 mM HEPES, pH 7.4, 300 mOsM) for 40 s, followed by 100 µM H_2_O_2_, freshly prepared in HEPES buffer directly added to the cells. The H_2_O_2_ influx was measured by following the time course of intracellular reactive oxygen species (ROS) accumulation upon the H_2_O_2_ challenge, reported as a first order rate constant obtained from the slope of a semi-logarithmic plot of fluorescence intensity vs. time [9].

### 2.6. Cell Migration

BxPC-3 cells were seeded in 12-well plates with an inoculum of 20,000 cells/cm^2^ and were allowed to grow till around 80% confluence. Then, a wound was scratched in the cell monolayer using a micropipette tip. After washing with phosphate buffered saline (PBS) pH 7.4, to remove cell debris, and adding fresh media with low FBS (2%), cells were incubated at 37 °C in a 5% CO_2_ incubator, and images of the wound closure were captured at 0, 12, and 24 h under a light microscope. The distance of the wound was measured using the software ImageJ (https://imagej.net accessed on 10 February 2022). Wound closure was normalized to original wound area at time 0 h [30].

### 2.7. Membrane Fluidity

BxPC-3 cells were cultured on µ-Slide 8 well IBIDI treated chambers (Ibidi, Gräfelfing, Germany) with 1 × 10^5^ cells. In the next day, the transfection was done as previously stated. After 48 h, medium was discarded and cells were washed twice with PBS. After that, PBS with 5 μM of Laurdan was added and the cells were incubated in a CO_2_ incubator at 37 °C for 20 min. Samples were examined on a Leica TCS SP5 inverted microscope (model DMI6000, Leica Microsystems CMS GmbH, Mannheim, Germany), with a 63× water (1.2-numerical aperture) apochromatic objective. Two photon excitation microscopy data were obtained with a Ti:sapphire laser (Mai Tai, Spectra-Physics, Darmstadt, Germany) as the excitation light source. The excitation wavelength was set to 780 nm and the fluorescence emission was collected at 400–460 nm and 470–530 nm to calculate the generalized polarization (GP) and obtain images based on this parameter. Whole image analysis was implemented in MATLAB (The Mathworks, Natick, MA, USA), with GP defined as GP = (I_400–460 nm_ − G × I_470–530 nm_)/(I_400–460 nm_ + G × I_470–530 nm_), where G is a calibration factor for the experimental setup. G was obtained by assessing Laurdan fluorescence in DMSO (GP_DMSO_ = 0.0357), using the same experimental conditions as those set for the measurements in living cells. Dark counts were subtracted to all intensity values. In the analysis, only Regions of Interest (ROI) corresponding to the plasma membranes in each cell were selected, restricting therefore the analysis to this cellular component. For each condition, at least 50 cells were analyzed.

### 2.8. Cell Elasticity

AFM studies were conducted using a force spectroscopy methodology previously described by us [31,32,33,34]. Differences in BxPC-3 cells elasticity between the studied conditions were evaluated by AFM-based force spectroscopy measurements. These measurements were performed in liquid environment, using the softest triangular cantilevers of OMCL TR-400-type silicon nitride AFM probes (Olympus, Tokyo, Japan), with a tip radius of approximately 15 nm and a resonance frequency of approximately 3 kHz in solution. The spring constants of the cantilevers were calibrated by the thermal fluctuation method, having a nominal spring constant close to 0.02 N/m. For every contact between the cells and the cantilever, the distance between them was adjusted to maintain a maximum applied force of 300 pN before retraction. For each condition tested, approximately 600 force vs. distance curves were collected. Data were analyzed to obtain the Young’s modulus, using JPK Image Processing software v. 6.055, by applying the Hertzian model. Values of AFM tip penetration depth into the cells were also obtained. Histograms of the forces curves for each studied group were constructed by choosing the ideal bin size to achieve the best fitted Gaussian model peak forces.

### 2.9. AFM Imaging

AFM studies were conducted using a NanoWizard IV atomic force microscope (JPK Instruments, Berlin, Germany) mounted on a Zeiss Axiovert 200 inverted optical microscope. BxPC-3 cells were cultured on 35 mm Petri dishes (TPP, Trasadingen, Switzerland) with 2 × 10^5^ cells. In the next day, the transfection was done as previously stated. After 48 h, medium was discarded and cells were washed twice with PBS. For imaging, cells were fixed with a 2% glutaraldehyde solution in PBS. The AFM head was equipped with a 15 μm z-range linearized piezoelectric scanner and an infrared laser. Imaging of the cells was performed in air, in tapping mode. Oxidized sharpened silicon tips (ACL, Applied NanoStructures, Inc., Mountain View, CA, USA) with a tip radius of approximately 6 nm, resonance frequency of about 60 kHz and spring constant of 3 N/m were used for the imaging. Imaging parameters were adjusted to minimize the force applied on the scanning of the topography of the cells. Scanning speed was optimized to 0.35 Hz and acquisition points were 512 × 512 pixels. Imaging data were analyzed with the JPK Image Processing software v. 6.055 (JPK Instruments). The area, volume, and elongation of each imaged cell were quantified using the SPIP software v. 6.6.0 (Image Metrology, Hørsholm, Denmark). Cell roughness was analyzed with Gwyddion software v. 2.55 (Czech Metrology Institute, Brno, Czech Republic) [31,35].

### 2.10. Cell–Cell Adhesion

BxPC-3 cells were cultured on 35 mm Petri dishes (TPP) with 2 × 10^5^ cells and treated as previously stated. On the day of the assay, 1 h before starting the cell–cell adhesion measurements, the culture medium was replaced by PBS in one of the Petri dish replicates, to ensure cell detachment. Afterwards, 5 min before the experiment, the culture medium of the other Petri dish replicate was replaced with serum-free medium. To conduct the AFM cell adhesion assays, tipless Arrow TL2 cantilevers (NanoWorld, Neuchâtel, Switzerland) with 1 µm thickness, 0.03 N/m spring constant, 7 kHz resonance frequency, 500 µm length and 100 µm width, were used. For this study, cantilevers were functionalized to attach one cell to the cantilever through cell capture with a semi-automatic approach system. For the functionalization, AFM cantilevers were cleaned with an intense UV light source for 15 min. Then, they were incubated in a 0.5 mg/mL biotinylated albumin solution at 37 °C, overnight, in a humidified incubator. Cantilevers were washed three times with PBS to remove unbound protein. After that, they were incubated in 0.5 mg/mL streptavidin and, finally, in 0.4 mg/mL biotin-concanavalin A (both incubations for 30 min, at room temperature). Functionalized cantilevers were mounted on the microscope. Then, a cell was captured by positioning the cantilever above the cell center and gently lowering it onto the cell for approximately 30 s. Applied force was adjusted to 300 pN before retraction. Data collection for each force-distance cycle was performed at 2.0 µm/s and with a z-length of 50 µm, using a JPK CellHesion module. Retract and extend delays of 5 s were applied. For each experimental condition, 3 cantilevers were used, with 2 cells being grabbed for each (total 6 cells at the top). With each cell at the top, glued to the cantilever, 6 cells were assessed in the Petri dish, with 5 force-distance curves per cell, giving a total of 36 cells analyzed per condition. Force-distance curves were analyzed using JPK Data Processing software v. 6.055. Values of the initial maximum detachment force, work (energy) necessary to overcome cell–cell adhesion (area under the curve on the force-distance plot), jumps, and membrane tethers were also obtained after analyzing the cell–cell adhesion retraction curves. We considered a membrane tether as a step-in force detected after a force plateau of more than 0.25 µm in distance, and jump events for force plateau of less than 0.25 µm in distance [36].

### 2.11. Cell Apoptosis

BxPC-3 cells were cultured on a 12-well plate with 1 × 10^6^ cells/well. The next day, transfection was carried out as previously stated. Cells were detached using trypLE (Thermo Fisher Scientific) and transferred with the complete medium into 2.0 mL tubes. Then, cells were centrifuged at 400× *g* for 5 min at 4 °C, and the supernatant was aspirated. Cells were washed and resuspended in 1 mL PBS. To each 1 mL of cell suspension, 1 μL of YO-PRO-1 (Thermo Fisher Scientific) and 1 μL propidium iodide (PI) stock solutions were added. Cells were incubated on ice for 20–30 min, protected from light. Three controls were used: unstained cells as a negative control, cells only with YO-PRO-1 as a single-color control, and cells only with PI also as single-color control. In addition to these controls, four samples (Control, siAQP3, siAQP5, and siAQP3/5) were analyzed according to manufacturer instructions. Samples were immediately analyzed by flow cytometry, with a BD Accuri C6 flow cytometer (Franklin Lakes, NJ, USA), using 488 nm excitation, with green fluorescence emission for YO-PRO-1 (530/30 nm bandpass) and red fluorescence emission for PI (610/20 nm bandpass), gating on cells to exclude debris. YO-PRO-1 and PI channel were measured and at least 10,000 events of single cells per sample were collected. Data was analyzed using FlowJo (Tree Star Inc., Ashland, OR, USA).

### 2.12. Statistical Analysis

All the experiments were performed in biological and technical triplicates. Results were expressed as the mean of at least three independent experiments. Statistical analysis between groups was performed by two-way ANOVA test, using Graph Prism software (GraphPad, La Jolla, CA, USA). *p* < 0.05 was considered as statistically significant.

## 3. Results

### 3.1. AQP3 and AQP5 Are the Most Abundant Aquaporins in BxPC3 Cells

**Figure 1:**
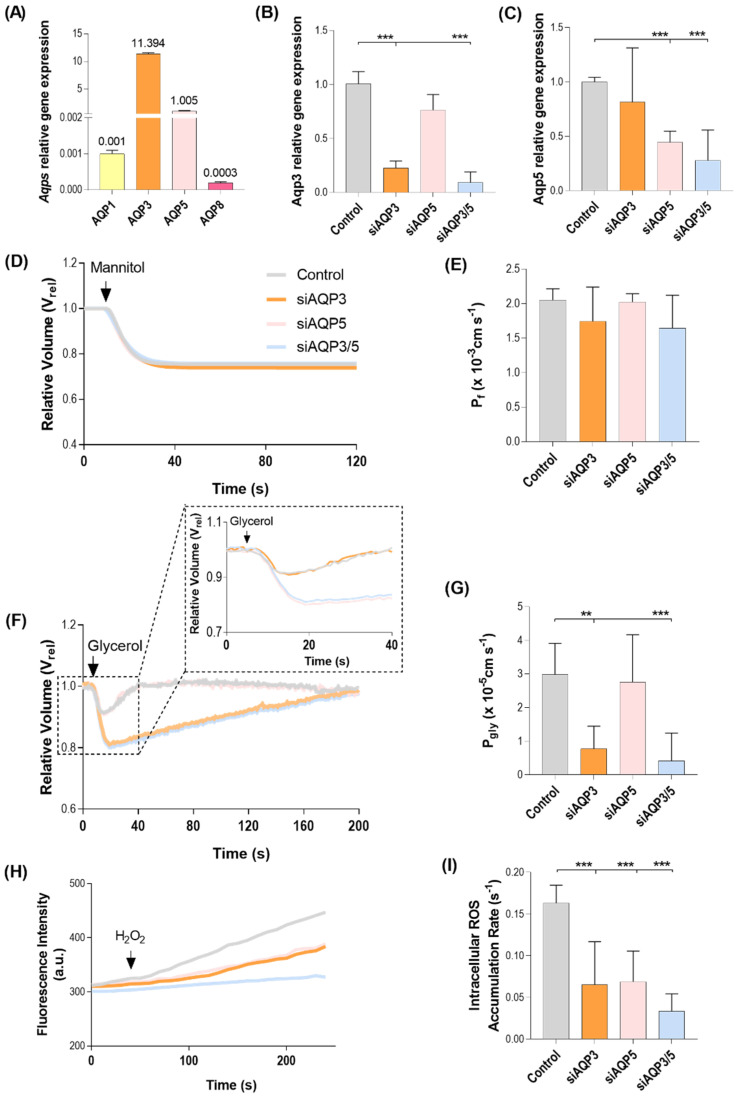
AQP3 and AQP5 are the most abundant AQPs in pancreatic BxPC-3 cells. (**A**) mRNA expression levels of the AQP paralogs naturally expressed in BxPC-3 cells. Values normalized to HPRT-1. (**B**–**G**) Validation of the loss-of-function model. (**B**,**C**) mRNA expression levels of BxPC-3 cells silenced for AQP3 (**B**) and AQP5 (**C**). Values normalized to HPRT-1. (**D**,**F**) Time course of cell volume changes caused by osmotic and solute challenges with mannitol (**D**) and glycerol (**F**) in control, siAQP3, siAQP5, and siAQP3/5 cells. (**E**,**G**) Water (P_f_) (**E**) and glycerol (P_gly_) permeabilities (**G**). (**H**) Representative traces of intracellular ROS accumulation, given by H_2_-DCFDA fluorescence increase after addition of 100 μM H_2_O_2_. (**I**) First-order kinetic rate constant of H_2_O_2_ influx through endogenous AQP3 and AQP5 in BxPC-3 cells after a 100 μM H_2_O_2_ challenge. Data represent a mean of *n* = 3 independent experiments ± standard error of the mean (SEM); *n* = 10 for permeability assays. ** *p* < 0.01; *** *p* < 0.001; both silenced vs. control cells; siAQP3, AQP3-silenced cells; siAQP5, AQP5-silenced cells; and, siAQP3/5, AQP3-, and AQP5-silenced cells.

A screening of AQPs paralogs expression was performed to validate their presence in BxPC-3 cells. AQP3 is the most abundant paralog in this cell line, followed by AQP5. AQP1 and AQP8 were also detected at low amounts (Figure 1A). AQP3 and AQP5 expression were transiently silenced using siRNA in around 75% (Figure 1B) and 50% (Figure 1C), respectively.

To validate the transfections and characterize AQP3 channel activity in BxPC-3 cells, plasma membrane permeability to water and glycerol were evaluated by fluorescence microscopy techniques. Water permeability calculated from the rate of cell volume equilibration of BxPC-3 cells exposed to a hyperosmotic mannitol solution was similar in non-silenced and silenced cells (Figure 1D,E). Glycerol permeability measured from the rate of cell volume re-equilibration after cell challenge with hyperosmotic glycerol solution, producing a fast cell shrinkage and cell re-swelling due to glycerol entrance followed by water (Figure 1F), revealed AQP3 as the main contributor to P_gly_, since AQP3-silenced cells displayed significantly reduced cell glycerol permeability (Figure 1G).

The transiently transfected BxPC-3 cells silenced for AQP3, AQP5, or AQP3/5 were tested to further investigate the biological relevance of their peroxiporin activity. The most representative AQPs in BxPC-3 cells, AQP3 and AQP5 (Figure 1A), are also H_2_O_2_ channels, as previously reported [9]. To further validate the gene downregulation effect at the protein level, the capacity for H_2_O_2_ permeation of AQP3-, AQP5-, and AQP3/5-silcenced cells was assessed by epifluorescence microscopy. The fluorescence signal of H_2_-DCFDA-loaded cells was monitored before and after the addition of 100 µM H_2_O_2_ (Figure 1H), and the H_2_O_2_ membrane permeation was evaluated indirectly by the intracellular accumulation of ROS (Figure 1I). As expected, the rate of H_2_O_2_ influx showed maximal values in control cells expressing both AQP3 and AQP5 peroxiporins. AQP3-silenced cells still expressing AQP5, and AQP5-silenced cells still expressing AQP3, revealed similar rates of fluorescence increase after the H_2_O_2_ challenge, and approximately 30% of that of the control cells (Figure 1I). In AQP3/5-silenced cells, a cumulative effect was observed, since H_2_O_2_ influx decreased to approximately 15% of the control due to the double silencing. Regarding AQP3 and AQP5 expression levels, despite similar H_2_O_2_ influx rates being measured in AQP3- and AQP5-silenced cells, our data suggest that AQP5 has a higher peroxiporin activity efficiency, resulting in an increased contribution to H_2_O_2_ membrane permeation.

### 3.2. Effect of AQP3- and AQP5-Silencing in Cell Migration

**Figure 2:**
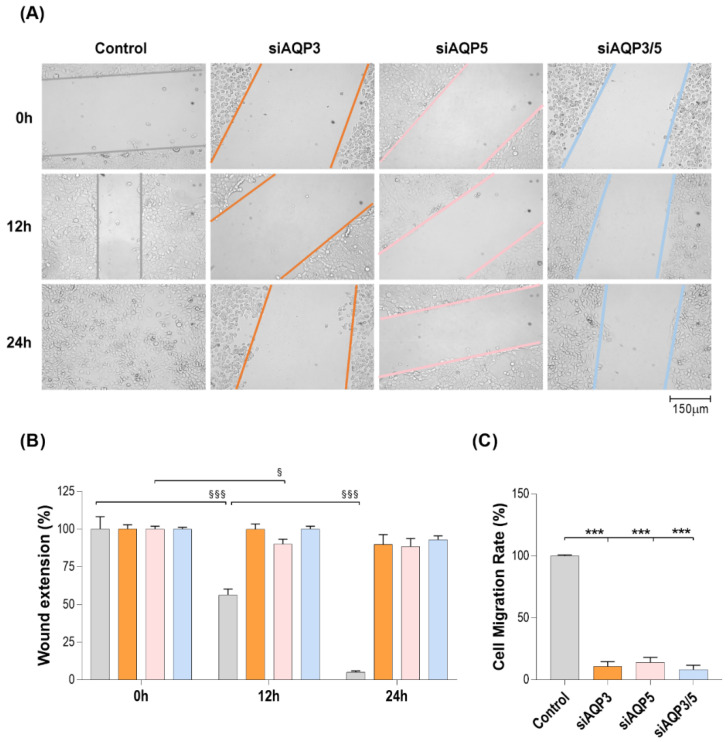
Effect of transient silencing of AQP3 and AQP5 in BxPC-3 migration. (**A**) Representative images of wound closure progression in control, AQP3-, AQP5-, and AQP3/5-silenced cells at 0, 12, and 24 h. (**B**) Wound extension progression in control, AQP3-, AQP5-, and AQP3/5-silenced cells at 0, 12, and 24 h past wound opening. (**C**) Cell migration rate of control, AQP3-, AQP5-, and AQP3/5-silenced cells. Results are expressed as mean ± SEM of three independent experiments. ^§^ *p* < 0.05; ^§§§^ *p* < 0.001; both times after wound opening vs. initial measurement (0 h); *** *p* < 0.001, silenced vs. control cells.

The relevance of AQP3 and AQP5 in cell migration and their implication in tumor progression was investigated in cells silenced for each paralog separately and simultaneously. AQP3-, AQP5-, and AQP3/5-silenced cells were followed during a wound closure assay, at 0, 12, and 24 h (Figure 2A). Impaired cell migration was already detectable in silenced cells 12 h after the wound scratch, when comparing to control cells. Differences became more evident 24 h after wound opening (Figure 2B). Our data demonstrate that the cell migration rate is strongly affected by the impaired expression of both AQP3 and AQP5 (11% and 14% of the control, respectively). The double silencing, siAQP3/5, resulted in a migration rate of 8% comparing to the control (Figure 2C).

### 3.3. AQP5 Influences Membrane Fluidity

**Figure 3:**
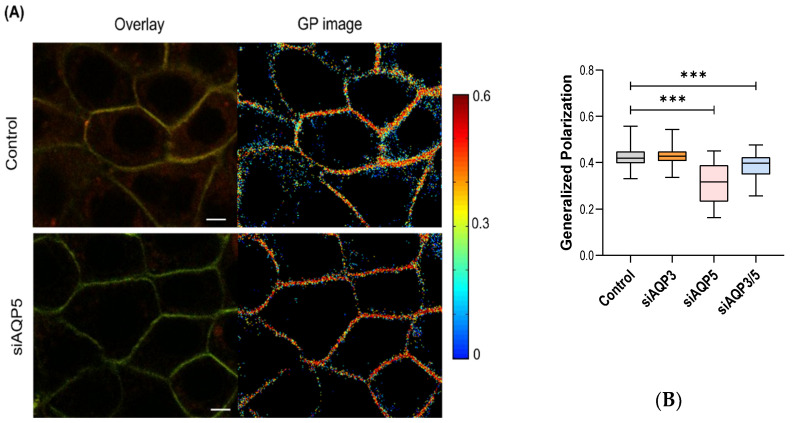
(**A**) Two-photon fluorescence microscopy of BxPC-3 cells labeled with Laurdan. Images from the left panel correspond to the overlay of equatorial z-section images taken with emission set at 400–460 nm (green channel) and 470–530 nm (red channel). GP images (right) were obtained by applying the GP function to the images from green and red channels, pixel by pixel. Scale bars: 5 μm. (**B**) GP values after incubation with Laurdan averaged from at least 50 different cells in each experimental condition for each one of the 3 independent experiments. *** *p* < 0.001, silenced vs. control cells.

As AQPs are membrane proteins, we sought to investigate if the silencing of AQP3 and/or AQP5 could be associated to changes in the organization of the plasma membrane of BxPC-3 cells. For that, we used the environment-sensitive fluorescent probe Laurdan, and two-photon excitation microscopy, which can detect variations in the ordering of the plasma membranes of living cells [37]. Changes in Laurdan fluorescence emission spectra are the result of alterations in the penetration of water molecules within the lipid bilayer. These changes can be quantified by calculating the generalized polarization. GP values can vary between 1 and −1. Higher GP values are associated to a less fluid plasma membrane and to a higher membrane ordering [38]. Laurdan GP measurements were performed for the four conditions under evaluation. Results show that silenced AQP5 and double-silenced cells presented a decrease in GP, meaning that these cells present a higher membrane fluidity (Figure 3). On the other hand, AQP3 silencing did not alter the GP, when compared with control cells.

### 3.4. AQP5 Promotes Changes in the Cell Biomechanical and Morphological Properties

**Figure 4:**
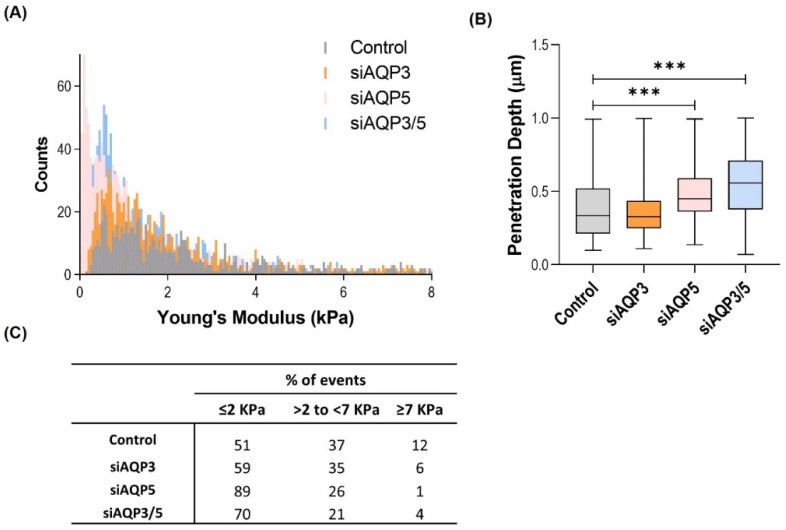
Biomechanical properties of BxPC-3 cells. (**A**) Young’s modulus. (**B**) Tip penetration depth into the cells. (**C**) Percentage of events at different Young’s modulus classes. Results were obtained in three independent experiments, with at least 600 force curves analyzed in each of them. *** *p* < 0.001, silenced vs. control cells.

The changes in cell membrane fluidity presented in the previous section prompted us to study possible alterations in the cellular biomechanical properties. From the AFM force-distance curves recorded during indentation experiments, we can determine the Young’s modulus and the AFM tip penetration depth of the BxPC-3 cells. Silenced AQP5 and silenced AQP3/5 cells showed more events at the lowest values of Young’s modulus, meaning that they are softer or less stiff than the other tested cells (Figure 4A,C). Corroborating these results, silenced AQP5 and double-silenced cells presented higher values of tip penetration depth for the same applied force (300 pN), indicating that these cells can deform more than the control cells (Figure 4B).

**Figure 5:**
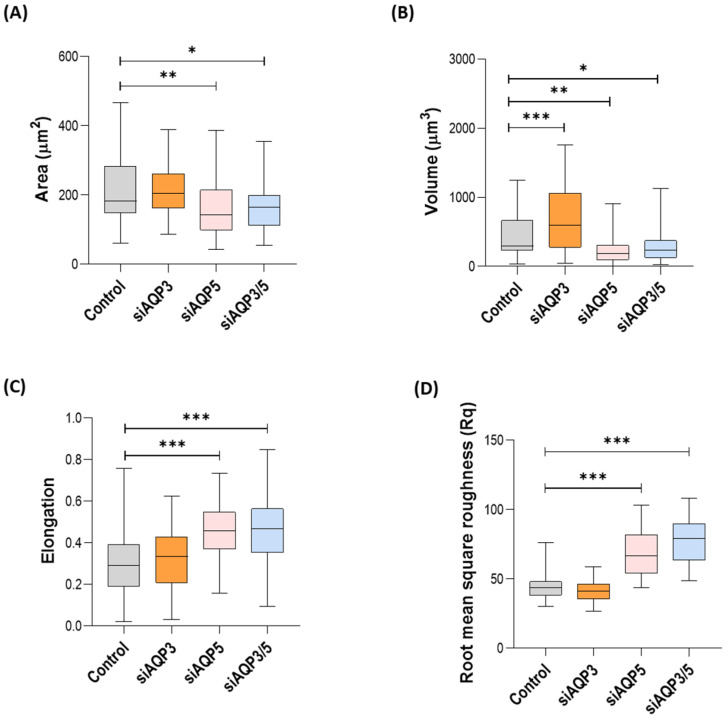
Morphological characteristics of the BxPC-3 cells under evaluation. (**A**) Cell area, (**B**) volume, (**C**) elongation (length of the shorter axis over the length of the longer axis), and (**D**) surface roughness. Results were obtained in three independent experiments, with at least 30 cells analyzed in each of them. * *p* < 0.05; ** *p* < 0.01; *** *p*<0.001; silenced vs. control cells.

Besides the differences in the biomechanical properties, we could also detect differences in morphological parameters, such as cell area, volume, elongation, and roughness. At the morphological level, the differences were again mostly dependent on AQP5 and almost independent of AQP3. Silenced AQP3 cells showed higher volume, when compared with control cells (Figure 5B), whereas silenced AQP5 and the double-silenced cells showed lower surface area, lower volume, higher membrane roughness, and were also more elongated than the control (Figure 5).

### 3.5. AQP3 and AQP5 Play an Important Role in Cell–Cell Adhesion

**Figure 6:**
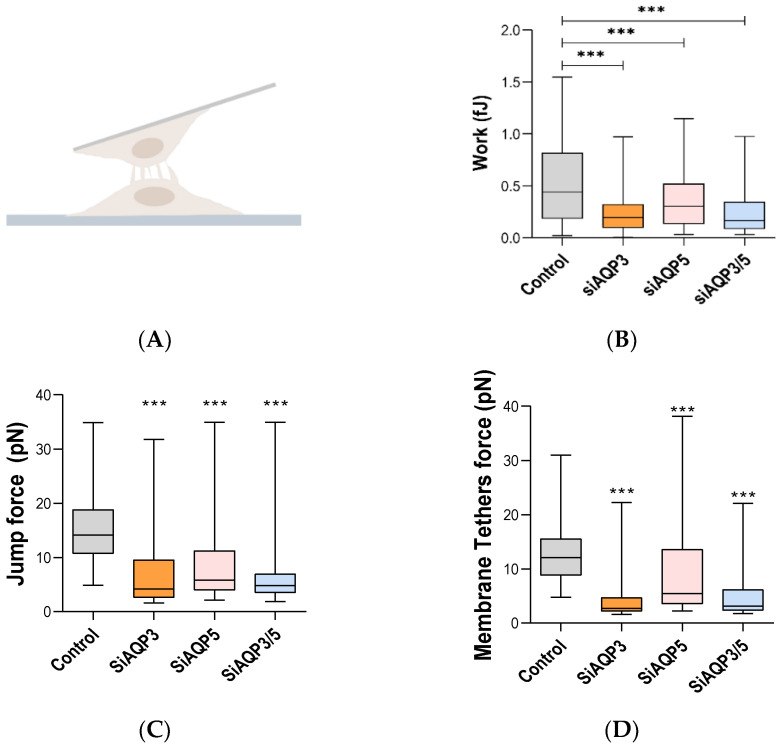
AFM cell–cell adhesion data. (**A**) Schematic representation of the interaction between a cell deposited on the surface of the solid substrate and another cell attached to a tipless AFM cantilever. (**B**) Work (energy) necessary to overcome cell–cell adhesion, detaching one cell from the other. (**C**) Jumps and (**D**) membrane tethers force data. Results were obtained in three independent experiments, with at least 60 force curves analyzed in each of them. *** *p* < 0.001, silenced vs. control cells.

Next, we investigated if cell–cell adhesion would be changed with the silencing of these aquaporins. We used AFM-based single cell force spectroscopy as an ultrasensitive method to quantitatively assess cell–cell adhesion under physiological conditions, probing the binding and subsequent unbinding of a single cell attached to the AFM cantilever and another cell on the Petri dish surface (Figure 6A). During AFM experiments, we performed several approach/retraction cycles between these cells. We found out that the work (energy) necessary to overcome cell–cell adhesion was lower in all the AQP-silenced cells, when compared with their control (Figure 6B). Moreover, we also analyzed the breaking of single interaction points, namely jumps (interactions involving receptors linked to the cytoskeleton) [39] and membrane tethers (events where a membrane tether was extruded before the breaking of a connection between surface components of two interacting cells) [36]. Cells with silenced AQPs showed lower jump and membrane tether events, when compared with control cells (Figure 6C,D). All cell–cell adhesion results agree on that not only AQP5, but also AQP3, plays an important role in this process.

### 3.6. Silencing AQP5 Induces Cell Apoptosis and Necrosis

**Figure 7:**
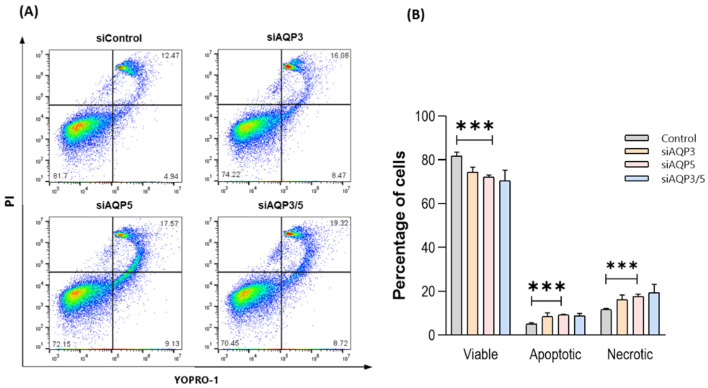
AQP5 is crucial for cell viability. (**A**) Cell viability analysis of control, siAQP3, siAQP5, and siAQP3/5 BxPC-3 cells stained with YO-PRO-1 and PI. Cells in the lower right quadrant are in early apoptosis, and those in the upper right quadrant are in mid and late apoptosis. (**B**) Percentage of viable, apoptotic, and necrotic cells. Results are expressed as mean ± SEM of three independent experiments. *** *p* < 0.001, silenced vs. control cells.

Some studies reported that a high expression of AQP5 can inhibit apoptosis [4,40]. Thus, we evaluated if silencing AQP3, AQP5, and AQP3/5 would promote apoptosis. Results showed that transfection with AQP5-siRNA significantly increased the percentage of apoptotic and necrotic cells, when compared with their control (Figure 7), indicating that the absence of AQP5 can lead to an increase in apoptosis and necrosis in this type of cells. No significant differences on cell viability were observed after silencing AQP3.

## 4. Discussion

Aquaporins are crucial for tissue homeostasis. In particular, the subgroup of peroxiporins is known to contribute to cell redox status, regulating several physiological processes such as cell migration and proliferation. Peroxiporin dysregulation destabilizes the finely regulated redox balance influencing the development of oxidative stress-related diseases, including cancer [8]. This work validated AQP3 and AQP5 as hydrogen peroxide channels in BxPC-3 cells, making them the major responsible for the intracellular H_2_O_2_ with external origin. Similar results have been previously described showing high efficiency in H_2_O_2_ transport of AQP5 comparing to AQP3 [9]. AQP3, an aquaglyceroporin, has been shown here for the first time to account for glycerol fluxes in pancreatic BxPC-3 cells. Interestingly, these cells present much faster glycerol permeation than other previously screened aquaglyceroporin-expressing human cells, such as human endothelial cells [41] and human primary monocytes [26], reflecting the important contribution of AQP3 for energy metabolism in BxPC-3 cells. Noteworthy, AQP3 high expression levels have been reported for different types of cancer, including skin [42], breast [43], and pancreatic [9] cancer, with demonstrated impact on cell migration and proliferation. Here, we observed that AQP3 and/or AQP5 silencing impair cell migration of BxPC-3 cells. Interestingly, AQP3 has been described as a key molecule in cell migration, mainly by modulating water fluxes at the edge of migrating cells, promoting lamellipodium formation [8,44]. Although proliferation might also be affected by AQP silencing, we assume that it has minor influence in migration assays. In fact, and considering the intrinsic variation of the physiological conditions for each cell culture, wildtype BxPC3 cells’ doubling time has been reported to be 36–72 h, depending on media supplementation with FBS [45,46,47,48], thus excluding proliferation contribution within the migration assay time course used here.

The assessment of hydration, fluidity, and ordering of the plasma membrane evaluated using the fluorescent probe Laurdan indicated that AQP5-silenced and double-silenced cells have lower values of GP, suggesting higher membrane hydration and higher membrane fluidity. The reduction in AQP3 alone did not affect the hydration at the intermediate levels of the bilayer. It is known that cancer cells have an increased amount of saturated lipids and higher ordering [49], which correlates well with a reduction in membrane fluidity and an increase in chemotherapy resistance [50]. In this context, we show, for the first time, that AQP5 contributes to membrane ordering in pancreatic cancer cells, rendering the membrane less fluid and possibly more prone to chemotherapy resistance. Furthermore, it is possible that the decrease in lipid packing corresponds to a higher permeability of the plasma membrane, and thus an increased intake of drugs through the cell membrane [51].

Recent studies have shown that, during cancer progression, alterations in cancer cells include changes on their biomechanical and morphological properties [52,53]. In fact, cell stiffness and adhesion have been considered crucial for cell transformation, invasion, and metastasis [54]. In this work, we found significant morphological alterations promoted by AQP5 silencing, namely a decrease in cell area and volume, and an increase in cell elongation, together with a higher surface roughness, when compared with control cells. On the other hand, silencing AQP3 seemed to interfere only with cell volume. In addition to these morphological changes, we also reported significant changes on cell stiffness. Cells with silenced AQP5 and double silencing were shown to be softer (or less stiff) than control, while no significant changes were observed in the cells with (only) AQP3 silenced. In fact, the viscoelastic and the morphological differences presented here were mostly dependent of AQP5 and almost independent of AQP3, indicating that AQP5 plays an important role on those cell properties.

Using AFM, we also assessed cell–cell adhesion. Our results show that the work (energy) necessary to overcome cell–cell adhesion was lower in AQP3 and/or AQP5 silenced cells. This indicates that the bonds between cells are weaker, or the number of connections established between them is lower than in the control. Further information is given by the significantly lower jump forces and membrane tethers on cells with silenced aquaporins, when compared with control cells. Jumps were associated to the unbinding of membrane ligand-receptor interactions without a preceding membrane deformation, while membrane tether events were associated with cell detachment with membrane invagination during the process [55]. AQP3 and/or AQP5 silencing was related with weak cell–cell adhesion, suggesting an involvement of AQPs in tumor metastases. Interestingly, comparing with early stages, in later stages of PDAC, an aggressive tumor that frequently form metastasis in liver [16], AQP5 expression is almost undetectable, corroborating our findings and underlining the AQP5 contribution to tumor aggressiveness.

Besides being a membrane channel, AQP5 can also have impact at the cytoskeleton level by directly binding to microtubules and increasing their assembly [56]. Therefore, we hypothesized that at least some of the differences presented above may be related to alterations in the cytoskeleton after AQP5-silencing. Cytoskeleton, a network comprised of actin filaments, intermediate filaments, and microtubules, is dynamically remodeled during cell migration, adhesion, proliferation, and differentiation [57]. Additionally, the cytoskeletal network is important for other structural and functional roles, such as maintaining cell morphology, signaling, and intracellular transport [58].

Using flow cytometry, we have also evaluated the level of apoptosis on these cells. Apoptosis is a conserved cellular process required for diverse biological functions, which is differentiated from necrotic cell death by specific highly conserved morphological and biochemical features [59,60]. Of those, one of the earliest morphological events is a pronounced cell shrinkage, termed apoptotic volume decrease [61], such as the detected for AQP5-silenced cells. In agreement with this, AQP5-silenced cells were also the ones that showed significant differences in apoptosis and necrosis, when compared with their control. Interestingly, in a previous report, AQP5-silenced human glioma cells also depicted an increased cell apoptotic rate comparing to control [62], corroborating our results.

Taken together, our findings demonstrate that the expression of AQP3 and AQP5 promotes alterations of the biological and biomechanical properties of these pancreatic cancer cells. To the best of our knowledge, this is the first demonstration that AQP3 is the major determinant for glycerol fluxes in BxPC-3 cells, and that AQP5 can cause significant changes in membrane ordering and fluidity in these cells, influencing chemotherapy resistance. Using atomic force microscopy, we provide insightful information on the implication of AQP3 and AQP5 expression in the biomechanical properties of BxPC-3 cells, namely cell area, cell volume, cell elongation, membrane roughness, cell stiffness, and cell–cell adhesion.

Although the results herein presented are clear and corroborated by gene expression and protein function, we cannot disregard the possible limitation of not having used a second independent siRNA to discard the possibility of off-target effects causing the functional responses observed. Additionally, extending these experiments to other cancer cell lines may bring confidence to the present results and clarify the general effect of AQPs over membrane biophysical properties. Further research is warranted to untangle the influence of AQP3 and AQP5 expression in the biophysical properties of cancer cells.

In a nutshell, the integration of biomechanical and bioimaging tools allow to infer the biological behavior of these cancer cells, suggesting AQP3- and AQP5-targeting as a promising strategy for anticancer therapy.

## Data Availability

Not applicable.
